# Potential Therapeutic Role for Apelin and Related Peptides in Diabetes: An Update

**DOI:** 10.1177/11795514221074679

**Published:** 2022-02-11

**Authors:** Ethan S Palmer, Nigel Irwin, Finbarr PM O’Harte

**Affiliations:** Diabetes Research Group, Ulster University, Coleraine, Northern Ireland, UK

**Keywords:** Apelin, apelin-13 analogues, diabetes, insulin secretion, glucose homoeostasis

## Abstract

Type 2 diabetes mellitus (T2DM) is an epidemic with an ever-increasing global prevalence. Current treatment strategies, although plentiful and somewhat effective, often fail to achieve desired glycaemic goals in many people, leading ultimately to disease complications. The lack of sustained efficacy of clinically-approved drugs has led to a heightened interest in the development of novel alternative efficacious antidiabetic therapies. One potential option in this regard is the peptide apelin, an adipokine that acts as an endogenous ligand of the APJ receptor. Apelin exists in various molecular isoforms and was initially studied for its cardiovascular benefits, however recent research suggests that it also plays a key role in glycaemic control. As such, apelin peptides have been shown to improve insulin sensitivity, glucose tolerance and lower circulating blood glucose. Nevertheless, native apelin has a short biological half-life that limits its therapeutic potential. More recently, analogues of apelin, particularly apelin-13, have been developed that possess a significantly extended biological half-life. These analogues may represent a promising target for future development of therapies for metabolic disease including diabetes and obesity.

## Introduction

In 2019, 463 million people were diagnosed with Type 2 diabetes mellitus (T2DM) worldwide^1^ and this epidemic is in a large part due to modern-day Westernised lifestyles that have been widely adopted globally.^2^ This includes consumption of high fat, energy dense and high carbohydrate foods, combined with sedentary lifestyles, leading to increased levels of obesity, hyperglycaemia, hyperinsulinaemia and insulin resistance.^3^ T2DM is characterised by impaired insulin secretion from pancreatic beta-cells and reduced insulin sensitivity of peripheral tissues, leading to overt hyperglycaemia.^4^ Under normal conditions, glycaemic control is maintained between 3.9 and 7.1 mmol/l glucose by a feedback loop connecting pancreatic islets and glucose metabolising, insulin sensitive tissues,^5^ which is associated with incretin hormone responses after a meal, but these pathways are impaired in T2DM contributing to hyperglycaemia. Ultimately, hyperglycaemia-induced excessive pancreatic beta-cell stimulation results in beta-cell dysfunction and a reduction in total beta-cell mass.^6^ Resulting elevated blood glucose concentrations, if untreated, can cause oxidative stress and inflammation, resulting in potential long-term damage to the micro and macro-vasculature leading to retinopathy, nephropathy, neuropathy and atherosclerosis.^7,8^ Taken together, these factors create a huge burden on healthcare systems and, in 2015, the estimated global cost of diabetes was $1.3 trillion, projected to increase to $2.1-2.8 trillion by 2030.^9^

Presently, very often the first-line treatment for T2DM is the biguanide metformin which primarily reduces insulin resistance and improves glucose metabolism.^10^ Unfortunately, failure of metformin over time often occurs, and metformin is used in combination with sulphonylureas which increase insulin output from pancreatic beta-cells.^11^ In addition, there are several other second and third line treatment options for T2DM including alpha glucosidase inhibitors, thiazolidinediones, sodium-glucose co-transporter-2 (SGLT2) inhibitors, incretin mimetics and enhancer drugs, as well as use of exogenous insulin injections.^12^ However, despite these numerous treatments, clinical management of T2DM and its associated comorbidities continues to cause problems in the management of this disease for many patients.^4^ This has led to heightened interest in diabetes therapies that not only manage the condition, but also counteract its associated complications. One potential option in this regard is apelin, an adipokine with established benefits on the cardiovascular system, as well as on energy homoeostasis and metabolism.

## Apelin

Apelin is an adipokine and the endogenous ligand of the APJ receptor.^13,14^ The APJ receptor is a G-protein coupled receptor (GPCR) first discovered through its sequence homology with the angiotensin II receptor by O’Dowd et al.^15^ It was deemed an orphan receptor until apelin was discovered as its cognate ligand following isolation from bovine stomach extracts in 1998.^16^ The APJ receptor is widely distributed throughout the body including skeletal muscle, cardiac muscle, pulmonary tissue, mammary glands, ovaries, brain, kidneys, pancreas and adrenal glands.^17^ Thus, indicating a role for apelin in a range of physiological processes including regulation of fluid homoeostasis,^18^ blood pressure^19^ and metabolic control.^20^ More recently, another endogenous ligand of the APJ receptor, termed ELABELA, was identified in human embryonic stem cells. ELABELA is a 32 amino acid peptide that appears to have a role in embryonic cardiac development.^21^

More recently, apelin has been identified as a potential therapeutic option for diabetes due to its ability to improve insulin production, insulin sensitivity and positively regulate diabetic complications, such as diabetes related kidney hypertrophy.^13,22^ Additionally, apelin has been demonstrated to induce satiety and reduce body weight,^23,24^ making it a very attractive target for future diabetes therapy. Coupled with its beneficial effects on the cardiovascular system,^25^ apelin may present a dual benefit in two related pathologies that are inter-linked. Indeed, prescribing effective diabetes medications, with additional cardiovascular benefits, represents a key objective of pharmaceutical care plans for many T2DM patients.^26^ The beneficial effects of apelin on the cardiovascular system are relatively well studied,^27,28^ whereas positive actions in diabetes less advanced. This review will focus predominantly on recent research on the therapeutic potential of apelin in diabetes.

## Apelin Processing

**Figure 1.**
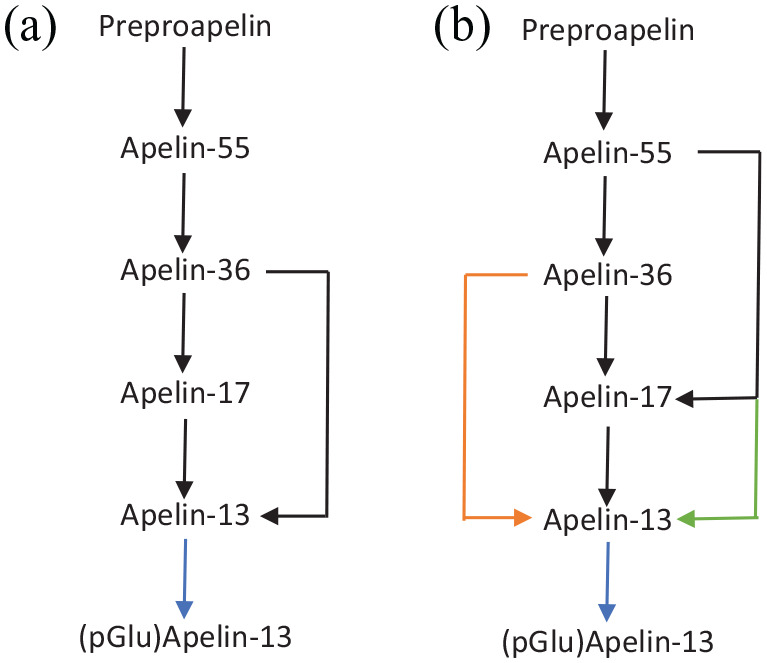
Hypothesised models of apelin processing. Two models are presented, with (a) depicting original philosophy and (b) an updated model based on recent literature. Blue arrows represent spontaneous N-terminal cyclisation of glutamine in apelin-13 to produce (pGlu)-apelin-13. Green arrows represent proprotein convertase subtilisin/kexin 3 (PCSK3) cleavage of apelin-55 to form apelin-13.^32^ Orange arrows represent apelin-36 processing to apelin-13 via as yet unidentified mechanisms.^31^

Apelin is initially translated from the APLN gene located on chromosome xq25-26.1 in humans, as the 77-amino acid preproapelin.^29^ An N-terminal 22-amino acid section is then proteolytically cleaved after Gly^22^ in the Gly^22^-Gly^23^ sequence forming apelin-55, referred to as proapelin. Initially, apelin-55 was considered a proprotein, and apelin-36 a precursor, for the more biologically active apelin-17 and apelin-13 isoforms (Table 1). This was due to the presence of Arg-Arg residues in apelin-36 which are typical of trypsin-like endopeptidase cleavage sites^30^ (Figure 1a). However, recent studies reveal that apelin processing is more complex than originally hypothesised. As such, apelin-55 is not a biologically inactive proprotein and it can activate the APJ receptor, although with less efficacy than its C-terminally derived shorter isoforms.^31^ Furthermore, the processes involved in the generation of apelin-13 from apelin-36 have yet to be fully elucidated,^32^ with (pGlu)apelin-13 representing the naturally occurring N-terminal cyclisation of the Gln^1^ residue in apelin-13. Indeed, Shin et al^33^ found that proprotein convertase subtilisin/kexin 3 (PCSK3) cleaves apelin-55 to produce apelin-13 without the formation of other isoforms, introducing a new pathway for apelin processing (Figure 1b).

**Table 1.** Primary amino acid sequences of apelin isoforms.

Peptide	Amino acid sequence
Apelin-77	MNLRLCVQALLLLWLSLTAVCGGSLMPLPDGNGLEDGNVRHLVQPRGSRNGPGPWQGGRRKFRRQRPRLSHKGPMPF
Apelin-55	GSLMPLPDGNGLEDGNVRHLVQPRGSRNGPGPWQGGRRKFRRQRPRLSHKGPMPF
Apelin-36	LVQPRGSRNGPGPWQGGRRKFRRQRPRLSHKGPMPF
Apelin-17	KFRRQRPRLSHKGPMPF
Apelin-13	QRPRLSHKGPMPF
(pGlu)apelin-13	(pGlu)RPRLSHKGPMPF
Apelin-12	RPRLSHKGPMPF

Amino acid notation of apelin peptide isoforms is denoted using standard single letter amino acid codes. pGlu is pyroglutamate.

## Apelin Structure/Function

Each isoform of apelin, namely apelin-55, apelin-36, apelin-17, apelin-13, (pGlu)apelin-13 and apelin-12 (Table 1), displays a random coil conformation, meaning they lack specific secondary structure and have no precise 3D shape.^31,34^ Nevertheless, each isoform appears to activate the APJ receptor in a similar fashion.^31^ This is explained by the relatively unique method through which apelin peptides bind to the APJ receptor. When bound to the receptor apelin peptides develop a curved conformation, binding at 2 different sites through both hydrophobic and polar interactions. Binding of the N-terminus causes the remainder of the peptide to fold approximately 90 degrees into a second binding site^35^ making it the only observation of a GPCR exhibiting dual-binding to a single endogenous ligand. Interestingly however, despite having similar receptor binding kinetics, the relative potency of apelin increases as its length decreases, with apelin-13, (pGlu)-apelin-13 and apelin-12 being the most biologically active isoforms, with relatively higher efficacy at the APJ receptor than the longer isoforms.^17^ Conversely, apelin-36 appears to bind to the APJ receptor for significantly longer than the shorter isoforms.^36^ This increase in receptor occupancy may be explained by the presence of additional negatively charged regions of the APJ receptor which interact with positively charged amino acids in the longer apelin-36 peptide.^35^ The APJ receptor primarily couples to Gα_i/o_, resulting in a range of downstream effects including inhibition of cAMP and ERK and Akt activation.^37^ Activation of the APJ receptor also increases intracellular Ca^2+^ though the precise mechanism has not been verified.^38^ However, this increase may be mediated through the PLCβ-PKC pathway via APJ coupling with either the Gβγ subunits of the Gα_i/o_ heterotrimer, Gα_q/11_ or both.^39^ In addition, it is believed that activation of the APJ receptor by shorter apelin isoforms leads to recruitment of both beta-arrestin-1 and -2, and the receptor is recycled to the cell surface within 60 minutes.^40^ In contrast, APJ receptor stimulation by apelin-36 results in beta-arrestin-1 activation with no receptor recycling observed after 4 hours.^40^ This may partly explain the differences in potency and biological effect of the different circulating apelin peptides.

## Brief Overview of Apelin Isoform Cardiovascular Benefits

Apelin-55 is the longest biologically active isoform of apelin and evokes a similar APJ receptor mediated ERK phosphorylation response as apelin-36 and apelin-17, but its therapeutic potential has yet to be fully elucidated.^32^ This may simply be a consequence of shorter apelin isoforms possessing comparable or enhanced bioactivity and thus being therapeutically more attractive. The beneficial effects of apelin-36 on the cardiovascular system are well established including protection against ischaemia and myocardial infarction.^25,41^ Apelin-36 has also been demonstrated to induce vasodilation^42^ alongside improving cardiac contractility.^43^ As discussed above, apelin-13 is one of the most potent apelin isoforms and thus has a range of positive cardiovascular effects.^44^ This includes antihypertensive actions as well as inducing notable improvements following myocardial infarction or heart failure,^45^ possibly linked to its action as an ionophore with increased cardiac contractility and output.^46^ pGlu-apelin-13 is the most common apelin isoform present in the human heart^47^ and exerts similar benefits as apelin-13 but has a longer half-life.^48,49^ Furthermore, apelin-12 exerts notable benefits on the cardiovascular system,^50^ with structural analogues of apelin-12 shown to inhibit cardiomyocyte damage and improve cardiac function in rats with left anterior descending coronary artery occlusion.^50^ In addition to this, non-peptigeric APJ receptor agonists have been generated that may facilitate oral administration. One such agonist, termed BMS-986224, has been shown to increase cardiac output in a rat model of cardiac hypertrophy and heart failure.^51^

## Apelin, Adipose Tissue and Insulin Resistance

Insulin resistance is a state where elevated circulating insulin concentrations are required to induce an appropriate glucose metabolising response in insulin sensitive tissues.^52^ Insulin resistance is commonly associated with obesity and can often result in the development of T2DM as pancreatic islets eventually fail to compensate for the increased insulin demand. Apelin is well documented to improve insulin action, but whether this is a result of direct tissue-mediated effects, indirect actions or a combination of both still needs to be fully established. However, as an adipokine, apelin plays an important role in adipose tissue function through a range of direct mechanisms. Apelin upregulates both brown adipose tissue (BAT) adipogenesis and white adipose tissue (WAT) browning.^53^ In isolated human and rat adipocytes, 100 nM pGlu-apelin-13 enhanced BAT adipogenesis through the upregulation of the adipogenic transcription factors C/EBPβ and PPARγ, whilst limiting the effects of TNFα, an inflammatory cytokine that inhibits BAT differentiation and induces BAT apoptosis.^53^ In the same study, C57BL/6 mice administered 1 µM/kg/day pGlu-apelin-13 for 12 days displayed reduced WAT weight whilst maintaining similar BAT mass compared to control mice, with no change in overall body weight between groups of mice. Interestingly, these pGlu-apelin-13 treated mice displayed elevated BAT markers in WAT, suggesting that apelin induces WAT browning, with similar observations in 3T3-L1 adipocytes.^53^ Taken together, the ability of apelin to induce BAT proliferation and WAT browning may provide a direct pathway through which apelin alleviates diabetic milieu.

Apelin has also been revealed to decrease fat storage in adipocytes, with apelin-13 (100 nM) shown to reduce lipid accumulation in 3T3-L1 adipocytes via PI3K-mediated upregulation of aquaporin-7, a glycerol transporter found in adipocytes.^54^ In full agreement, 1 μM pGlu-apelin-13 treatment for 10 to 14 days was demonstrated to inhibit differentiation of 3T3-L1 pre-adipocytes to lipid accumulating mature rounded adipocytes.^55^ Indeed, more recently apelin has been confirmed to inhibit adipogenic differentiation and promote lipolysis.^56^ Additionally, apelin can improve lipid metabolism in other metabolically active tissues, with 0.2 μmol/kg/day apelin-13 administration improving lipid utilisation and insulin resistance, via improved mitochondrial oxidative capacity and enhanced fatty acid oxidation, in soleus muscle of insulin resistant high fat fed C57BL/6 mice.^57^ Similarly, i.p. administration of 0.1 μmol/kg/day pGlu-apelin-13 for 28 days improved hepatic lipid metabolism and reduced steatosis in high fat fed mice.^58^

## Diabetes

Benefits of apelin on insulin action would suggest obvious therapeutic potential for diabetes, but some dispute regarding direct effects of apelin on the endocrine pancreas have caused some confusion in this regard. As such, an early study conducted by Sörhede Winzell et al^59^ demonstrated that intravenous administration of apelin-36 (2 nmol/kg body weight) inhibited acute glucose stimulated insulin secretion in both lean control and high fat fed obese C57BL/6 mice. In the same study, 1 µmol/l apelin-36 also inhibited insulin production from isolated murine islets under stimulatory and non-stimulatory glucose conditions. A further study examined the *in vitro* effect of (pGlu)apelin-13 on insulin secretion from rat insulinoma INS-1 cells, revealing that (pGlu)apelin-13 (10-1000 nmol/l) had no insulinotropic effects at lower glucose concentrations and inhibited insulin secretion by up to 40% at 10 mmol glucose.^60^ It was determined that this inhibitory effect was due to the PI3-kinase activation of phosphodiesterase 3.^60^

Despite these initial observations of decreased insulin output induced by apelin, subsequent studies in streptozotocin-induced (T1DM model) and high fat fed diabetic (T2DM model) rats revealed highly beneficial effects of apelin-13 (100 nmol/kg bw) including improvement of insulin sensitivity and reduction of plasma glucose after daily administration for 2 weeks.^61^ In addition, a 200 pmol/kg body weight i.v. injection of apelin-13 was also demonstrated to improve acute glucose tolerance in normal and high fat fed rats following an oral glucose tolerance test.^61^ The same study noted that 45 minutes incubation of apelin-13 (10-1000 nM) improved glucose utilisation in insulin sensitive soleus muscle. These results have been replicated in more recent studies that reveal daily i.p. administration of apelin-13 (0.1 µmol/kg bw) for 6 weeks improved insulin sensitivity and reduced blood glucose concentrations in high fat fed diabetic rats (T2DM model).^62^ Furthermore, (pGlu)apelin-13 has also been shown to dose-dependently increase glucose uptake in ex vivo human adipose tissue via 5' adenosine monophosphate-activated protein kinase (AMPK) phosphorylation.^63^ In agreement, apelin-13 has previously been shown to improve glucose tolerance in normal mice as well as augment glucose uptake in insulin-resistant high fat fed mice.^64^ Similar observations have been made following intracerebroventricular injection of (pGlu)apelin-13 in high fat fed mice,^65^ that are considered to be dependent on nitric oxide production.^65^

In keeping with this, apelin deficient mice present with insulin resistance that is exacerbated by feeding a high-fat diet.^66^ However, daily infusion of (pGlu)apelin-13 for 4 weeks (2 mg/kg bw) in these mice, via a subcutaneous osmotic pump, reversed insulin resistance.^66^ This phenomenon was also observed following similar (pGlu)apelin-13 administration in obese diabetic *db/db* mice (T2DM model) that do not have a genetic apelin deficiency.^66^ Interestingly, a study conducted by Han et al^67^ revealed that deletion of the APJ receptor in the pancreatic islets of C57BL/6 mice augments glucose stimulated insulin secretion, implicating a positive role for apelin in glucose stimulated insulin production. Additionally, pancreatic APJ receptor deletion in C57BL/6 mice caused a significant reduction in islet size, area, and beta-cell function. Glucose clearance was also impaired by specific pancreatic APJ deletion, highlighting the important role of the apelinergic system in pancreatic islet function and overall glucose homoeostasis. Moreover, apelin treatment (precise isoform was not stated) of cultured rat insulinoma cells also increased beta-cell growth, indicating that apelin may play a positive role in beta-cell proliferation.^67^ To date, only one study into the effect of apelin treatment in diabetes has been conducted in humans.^68^ In this regard, 16 non-diabetic overweight men (BMI 25-30 kg/m^2^) were subjected to a 24 h hyperinsulinaemic-euglycaemic clamp and administered either 9 or 30 nmol/kg bw of (pGlu)apelin-13.^68^ Interestingly, the 30 nmol/kg bw dose significantly increased insulin sensitivity, corroborating previously reported apelin induced improvements of insulin sensitivity in animal models.

Taken together, although apelin appears to have clear antidiabetic efficacy, there are conflicting reports regarding the underlying mechanisms. In brief, apelin clearly improves insulin sensitivity, reduces blood glucose and increases glucose utilisation.^61-68^ However, contrasting observations on the effect of apelin on insulin secretion have been documented.^59,60^ Such discrepancies may be explained by the different apelin isoforms and experimental systems employed in these studies (Table 2). The diversity in findings might indicate that different apelin isoforms, despite activating the APJ receptor, produce distinct tissue specific effects. However, despite these discrepant findings, there is a general consensus that apelin isoforms improve metabolic status, suggesting clear potential for diabetes therapy.

**Table 2.** Overview of the effect of various apelin peptides on glycaemic control and metabolism.

Isoform	Effects on glycaemic control and metabolism
Apelin-36	Reduces insulin secretion in mice^54^
	Reduces insulin secretion in vitro in hyperglycaemic conditions^54^
Apelin-13	Reduce blood glucose in normal mice, high fat fed mice, high fat fed rats and streptozotocin induced diabetic rats^57^
	No change in plasma insulin in obese-diabetic mice^57^
	No change in serum insulin concentration in streptozotocin induced type 1 diabetic rats^57^
	Improves glucose tolerance and utilisation in normal and high fat fed mice and high fat fed rats^58^
(pGlu)-apelin-13	Increases glucose uptake in ex vivo human adipose tissue^58^
	No effect on insulin secretion at 3 mmol/L glucose, inhibition in insulin secretion and cAMP at 10 mmol/L glucose^55^
	Improves insulin sensitivity in apelin knockout and obese diabetic mice^59^
	Improves insulin sensitivity in overweight men^61^

Major effects of each apelin isoform on glycaemic and metabolic control. The associated citations are also included.

## Diabetic Complications

In terms of diabetes-induced kidney dysfunction and inflammation, twice daily administration of apelin-13 (400 pmol/kg bw) for 9 weeks was shown to impede development of both conditions in Akita mice (T1DM model). This included prevention of increased kidney size, as well as reduction in glomerular filtration rate and proteinuria, mediated via an inhibitory effect on histone acetylation, which is associated with a range of diabetic vascular complications.^69^ Notably, these are classical symptoms of diabetic nephropathy which affect approximately one third of diabetic patients.^70^ Further independent observations found similar benefits with (pGlu)apelin-13 in male FVB/Ove26 mice, a genetically-induced model of Type 1 diabetes, to reduce diabetes induced kidney and glomerular hypertrophy, as well as renal inflammation and albuminuria.^71^ Furthermore, these mice exhibited reduced renal APJ receptor expression, with 2 weeks daily subcutaneous (pGlu)apelin-13 treatment (150 µg/kg bw) counteracting this, suggesting that the apelinergic system plays an important protective role within the kidneys.^71^ Furthermore, reduced ELABELA levels may contribute to increased risk of diabetic kidney disease.^72,73^ A study conducted in 80 patients found that serum ELABELA levels were negatively correlated with urinary albumin creatinine ratio.^74^ In this study, patients with the lowest serum ELABELA levels had macroalbuminuria and elevated serum creatinine which may lead to kidney disease.^74^ An additional study conducted in 50 healthy and 100 diabetic patients found a similar negative correlation between serum ELABELA and urinary albumin creatinine ratio.^75^

As documented above, the beneficial role of apelin in cardiovascular disease is well established.^24,25,76,77^ Recent research has shown that apelin may also protect against diabetes induced cardiac hypertrophy. Daily apelin-13 treatment for 8 weeks (100 nmol/kg bw) significantly improved both cardiac and left ventricular indices in male Wistar streptozotocin-induced diabetic mice (T1DM model).^78^ This improvement was mirrored by a decrease in left ventricular extracellular matrix protein deposition and collagen levels which are key risk factors in cardiac hypertrophy, with additional benefits for hyperglycaemia and glucose tolerance.^78^ Corresponding benefits of i.p. apelin-13 (200 µg/kg bw for 4 weeks) on cardiac function and metabolic status have also been observed in obese diabetic Goto-Kakizaki (GK) rats (T2DM model).^79^ Such benefits may be linked to apelin-induced sphingosine kinase 1 mediated inhibition of transforming growth factor beta (TGF-β).^80^ Thus, TGF-β is a proinflammatory cytokine that is upregulated in diabetes^81^ and induces cardiac fibrosis alongside extra cellular matrix protein deposition,^82^ representing key risk factors in cardiac hypertrophy.

## Apelin Analogues and Diabetes

Recently, various laboratories have created modified apelin analogues to generate APJ receptor activators with increased half-life and enhanced antidiabetic effects. Additionally, analogues of apelin have been developed to target cardiovascular disease, with reports of beneficial effects on myocardial dysfunction and hypertension in rats.^83,84^ These molecules have been reviewed in detail elsewhere,^77,85^ and the current report will focus on apelin analogue advantages in diabetes. The earliest study to utilise apelin analogues in diabetes therapy employed an adeno-associated virus minigene system to mimic systemic expression of apelin-13, apelin-36 and modified analogues of apelin-36, namely apelin-36(L28A) where Leu^28^ was substituted by Ala.^28,86^ BDF mice were administered 5 × 10^11^ genome copies of an apelin-expressing adeno-associated virus via tail vein injection and placed onto a high-fat diet for 8 weeks. Metabolic status was then measured periodically. Although apelin-36(L28A) displayed a 100-fold reduction in APJ activation potency, it reduced body weight and blood glucose to a greater extent than native apelin-36 and improved glucose tolerance.^86^ Thus, suggesting that minor modifications to the sequence of apelin may yield improved antidiabetic actions presumably through prolongation of the peptide half-life. A non-peptidergic, organic, pyrazole-based APJ receptor agonist, named Compound 13, has also been generated.^34^ Compound 13 reduced food intake and body weight in high fat fed mice, whilst additionally improving glucose tolerance and reducing liver steatosis.^34^

Further preclinical work in the field of diabetes has been conducted with well characterised analogues of apelin-13. As such, 9 apelin-13 analogues were generated and characterised in the laboratory of O’Harte et al^38^ (Table 3). All peptide analogues displayed an increased biological half-life, but elicited differing effects on insulin secretion, with 5 stimulating insulin secretion, 3 inhibiting insulin secretion (thought to be APJ receptor antagonists) and 1 producing no significant effect (Table 3). These observations were mirrored by respective increases and decreases in in vitro cAMP and intracellular Ca^2+^ concentrations in pancreatic BRIN-BD11 cells^38^ (Table 3). Interestingly, bioactivity profiles of the apelin-13 analogues at the level of the pancreatic beta-cell were directly reflected by the ability of each analogue to inhibit food intake.^38^ The 2 most promising lead compounds, apelin-13 amide and (pGlu)apelin-13 amide, were further examined for their antidiabetic efficacy in high fat fed diet induced obese (DIO) diabetic male NIH Swiss mice (T2DM model).^87^ Twice daily i.p. administration of both analogues for 28 days at 25 nmol/kg bw reduced fasting blood glucose, feeding, circulating triglycerides and body weight with an increase in plasma insulin and improved glucose tolerance^87^ (Table 4). (pGlu)apelin-13 amide also increased energy expenditure and pancreatic insulin content whilst reducing total body fat and LDL-cholesterol, potentially making it a useful therapeutic target agent for diabetes therapy^87^ (Table 4).

**Table 3.** Overview of acute effects of modified apelin-13 analogues on metabolic status in mice.

Modified apelin-13 analogue	In vitro insulin secretion (ng/10^6^ cells/20 min)	Ca^2+^ flux (RFU)	Intracellular cAMP (pM/ml)	AUC acute plasma insulin (mmol/l.min)	AUC acute glucose tolerance (mmol/l.min)	AUC 16 h delayed tolerance glucose (mmol/l.min)	AUC 16 h delayed plasma insulin (mmol/l.min)	Acute food intake (g)
(Ala^13^)apelin-13	↓	↓	↓↓	↓	↑	ND	ND	ND
(Val^13^)apelin-13	↓↓↓	↓	↓↓	↓↓	↑↑	ND	ND	ND
(Tyr^13^)apelin-13	↑↑↑	↑↑	↑	↑	↓	ND	ND	ND
Apelin-13-amide	↑↑↑	↑↑	↑↑	↑↑↑	↓↓	ND	ND	ND
(pGlu)apelin-13	↑↑	↑	↑	–	–	ND	ND	ND
pGlu(Ala^13^)apelin-13	–	↓	↓↓	↓	↑	ND	ND	ND
pGlu(Val^13^)apelin-13	↓↓↓	↓	↓↓	↓↓	↑	ND	ND	ND
pGlu(Tyr^13^)apelin-13	↑↑↑	↑↑	↑	↑↑	↓	ND	ND	ND
(pGlu)apelin-13-amide	↑↑↑	↑↑↑	↑	↑↑	↓↓	ND	ND	ND
(Lys^8^GluPAL)apelin-13-amide	↑↑↑	↑↑↑		–	–	↑↑	↑↑↑	↓↓↓
Lys^8^GluPAL(Tyr^13^)apelin-13	↑↑	↑	–	–	–	–	↑	–
Lys^8^GluPAL(Val^13^)apelin-13	↓↓↓	–	↓↓	ND	ND	ND	ND	↑↑
pGlu(Lys^8^GluPAL)apelin-13 amide	↑↑↑	↑↑↑	↑	↑	↑	↑↑	↑↑↑	↓↓↓

Abbreviation: ND, not determined

Insulin secretion, Ca^2+^ flux and intracellular cAMP was measured in BRIN-BD11 cells following apelin-13 analogue administration at 10^−12^ to 10^−6^ M. Acute and delayed plasma insulin and glucose tolerance AUC values were measured in high fat fed DIO mice following 25 nmol/kg body weight apelin-13 analogue and 18 mmol/kg body weight glucose administration and calculated using GraphPad Prism ver. 5.0. Acute food intake was measured in male NIH Swiss mice trained to eat during a 3 hour period, mice were injected immediately prior to food being available 25 nmol/kg body weight) with food intake measured at 30 minutes intervals.

- represents no significant change, ↑/↓ represents *P* > .05, ↑↑/↓↓ represents *P* > .01 and ↑↑↑/↓↓↓ represents *P* > .001 increase/decrease vs glucose control in each study.

**Table 4.** Overview of longer-term beneficial effects of various apelin-13 analogues in rodent models of obesity-diabetes.

Modified apelin-13 analogue	Body weight (g)	Energy intake (Kj/g)	Blood glucose (mM)	Plasma insulin (ng/ml)	AUC oral glucose tolerance (mmol/l.min)	AAC insulin sensitivity (mmol/l.min)	HbA1c (mmol/mol)	Circulating triglycerides (mmol/l)	LDL cholesterol (mmol/l)
Apelin-13 amide	↓	↓	↓↓	–	↑↑	–	–	↓	–
(pGlu)apelin-13 amide	↓	↓	↓↓	↑↑	↑↑↑	↑	↓	↓	↓↓
(Lys^8^ GluPAL)apelin-13	↓	↓↓	↓↓↓	↑↑	↑↑↑	↑↑	↓	↓	↓
pGlu(Lys^8^ GluPAL) apelin-13 amide	↓↓	↓↓↓	↓↓	↑↑	↑↑↑	↑↑	↓↓	↓↓↓	↓↓

Comparative effects of twice daily administration of apelin-13 amide and (pGlu)apelin-13 amide (25 nmol/kg bw) for 28 days, as well as once daily (Lys^8^GluPAL)apelin-13 and pGlu(Lys^8^GluPAL)apelin-13 amide (25 nmol/kg bw) for 40 days in DIO obese mice. Body weight, energy intake, plasma glucose and insulin were measured at regular intervals throughout both studies. Glucose tolerance was assessed in response to administration of 18 mmol/kg glucose and insulin sensitivity following i.p. injection of exogenous insulin, at the end of the respective studies. Terminal analyses included measurement of HbA1c using a point-of-care A1CNow+ kits and lipid profile determined using an I-Lab 650 clinical analyser.

- represents no significant change, ↑/↓ represents *P* > .05, ↑↑/↓↓ represents *P* > .01 and ↑↑↑/↓↓↓ represents *P* > .001 increase/decrease versus saline treated DIO control mice in each study.

An additional 4 fatty acid derived analogues of apelin-13 with acylation at Lys^8^ alongside additional N- and C- terminal modifications have been generated including (Lys^8^GluPAL)apelin-13 amide, pGlu(Lys^8^GluPAL)apelin-13 amide, Lys^8^GluPAL(Tyr^13^)apelin-13 and Lys^8^GluPAL(Val^13^)apelin-13^88^ (Table 3). The fatty acid adduct was employed to extend biological half-life, and indeed each acylated apelin-13 analogue exhibited increased in vitro half-life compared to native apelin-13 (24 hours vs >2.1 hours), with further investigations revealing that Lys^8^GluPAL(Tyr^13^)apelin-13 had an in vivo half-life of 2.5 to 3 hours.^88^ (Lys^8^GluPAL)apelin-13 amide, pGlu(Lys^8^GluPAL)apelin-13 amide and Lys^8^GluPAL(Tyr^13^)apelin-13 demonstrated dose dependent insulin secretory actions, with Lys^8^GluPAL(Val^13^)apelin-13 causing a dose dependent inhibition in both isolated islets and cultured BRIN-BD11 cells.^88^ Additionally, (Lys^8^GluPAL)apelin-13 amide and pGlu (Lys^8^GluPAL)apelin-13 amide positively augmented glucose uptake in 3T3-L1 adipocytes and reduced food intake in high fat fed diet induced obese (DIO) diabetic male NIH Swiss mice (T2DM model).^88^ The most effective analogue in this study appeared to be pGlu(Lys^8^GluPAL)apelin-13 amide which increased in vitro and in vivo insulin secretion, improved glucose tolerance and reduced food intake^88^ (Table 4).

More notably, lead analogues from these studies induced similar antidiabetic effects in DIO diabetic male NIH Swiss mice (T2DM model), highlighting a potentially key therapeutic advantage due to their prolonged duration of action, thereby necessitating fewer daily injections.^88^ In keeping with this, pGlu(Lys^8^GluPAL)apelin-13 amide and (pGlu)apelin-13 amide have been shown to promote important antidiabetic actions in *db/db* mice (T2DM model),^89^ confirming their effectiveness across a range of diabetes aetiologies. Furthermore, pGlu(Lys^8^GluPAL)apelin-13 amide was recently shown to reduce beta- to alpha-cell transdifferentiation and beta-cell apoptosis in streptozotocin-induced diabetic mice (T1DM model), highlighting additional potentially important antidiabetic promise in terms of preserving pancreatic islet morphology.^90^

## Conclusion

Of the established apelin isoforms, apelin-13 appears to be the most interesting in terms of diabetes therapy owing to its ability to improve glucose tolerance and insulin sensitivity, as well as potential effects to enhance insulin secretion. Apelin-13 administration also appears to decrease body weight, likely by directly reducing food intake alongside increasing BAT mass and activity. Importantly, apelin-13 also improves diabetic complications including diabetic nephropathy and cardiac hypertrophy. The short biological half-life of native apelin-13 precludes therapeutic utility; however, analogues of apelin-13 have been generated that possess significantly increased in vivo half-lives whilst retaining key antidiabetic actions. These apelin-13 analogues improve insulin sensitivity, glucose tolerance, glycaemic control, lipid profile and plasma insulin whilst also inducing satiety and reducing body weight. Notably, related benefits on pancreatic islet architecture could suggest disease-modifying effects of these apelin-13 analogues. Therefore, such apelin analogues, and particularly longer-acting forms such as pGlu(Lys^8^GluPAL)apelin-13 amide have exciting potential in the future of diabetes therapy.
